# Supplementary material for: Identification of photoperiod-induced specific miRNAs in the adrenal glands of Sunite sheep (Ovis aries)
Source: Front Vet Sci. 2022 Jul 22;9:888207. doi: 10.3389/fvets.2022.888207 (PMC9354845; doi:10.3389/fvets.2022.888207)
Supplement: Supplementary file 1 [file Data_Sheet_1.ZIP › Supplementary file/miRDeep2 file.zip!/miRDeep2 file/Know_Structure/SP42A3/oar-mir-134.pdf]

miRBase precursor : oar-mir-134
Total read count : 51
oar-miR-134-5p read count : 45
oar-miR-134-3p read count : 5
remaining reads : 1

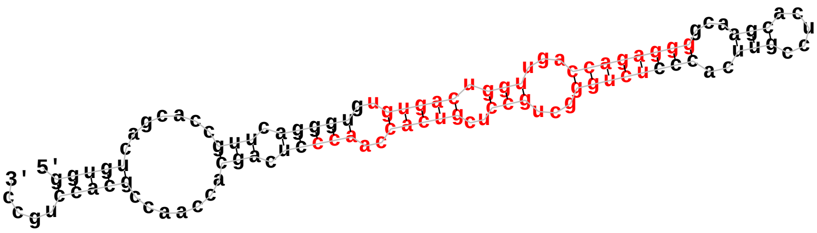

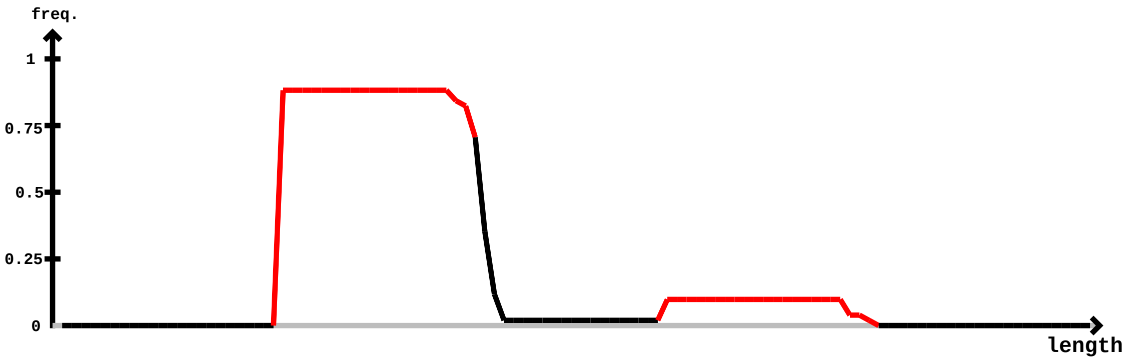

oar-miR-134-3p

oar-miR-134-5p

```
5'- ggugucagcaccguucagggugugugacugguugaccagagggcaagcacuccguucacccucugggcugccucgucaccaacccucagcaccaaccgcaccugcc   -3'    exp
    (((((.......(((.(((((..(((((.(((...((((((((...(((.....)))..))))))))...)))..)))))..))))).))).......)))))....   reads      mm        sample
    ......................ugugacugguugaccaga...................................................................   2          0         seq
    ......................ugugacugguugaccagag..................................................................   1          0         seq
    ......................ugugacugguugaccagagg.................................................................   6          0         seq
    ......................ugugacugguugaccagaggg................................................................   19         0         seq
    ......................ugugacugguugaccagagggg...............................................................   12         0         seq
    ......................ugugacugguugaccagaggggc..............................................................   5          0         seq
    ...........................................gcaagcacuccguucacc..............................................   1          0         seq
    .............................................................cucugggcugccucgucacc..........................   1          0         seq
    ..............................................................ucugggcugccucgucacc..........................   2          0         seq
    ..............................................................ucugggcugccucgucaccaa........................   1          0         seq
    ..............................................................ucugggcugccucgucaccaac.......................   1          0         seq
```
